# Arabic Coffee Consumption and Its Correlation to Obesity Among the General Population in the Eastern Province, Kingdom of Saudi Arabia

**DOI:** 10.7759/cureus.30848

**Published:** 2022-10-29

**Authors:** Rawan A Alawadh, Naushad Abid, Aeshah S Alsaad, Hussain I Aljohar, Meshal M Alharbi, Fai K Alhussain

**Affiliations:** 1 Internal Medicine Department, College of Medicine, King Faisal University, AlAhsa, SAU; 2 Rheumatology, King Faisal University, AlAhsa, SAU; 3 Neurology, King Faisal University, AlAhsa, SAU

**Keywords:** arabic coffee, coffee, consumption, general population, obesity

## Abstract

Aim

This study aims to assess the relationship between Arabic coffee consumption and obesity among the Saudi adult population.

Subjects and methods

This is a cross-sectional study conducted among the population living in the Eastern Province of Saudi Arabia. A self-administered questionnaire was distributed among the general population using an online survey. The questionnaire included socio-demographic characteristics, anthropometric measurements, and different parameters to assess the factors associated with Arabic coffee consumption.

Results

Three hundred eighty-nine participants were involved (57.1% females vs 42.9% males). Obese respondents constitute 33.7%. More than half of the total (53.5%) drink coffee daily. Our results revealed that there were statistically significant associations between the overall level of BMI according to the frequency of drinking coffee per day (p<0.001), number of cups drank per day (p=0.006), favorite additives for coffee such as milk (p=0.017), cardamom (p=0.017), other calorie additives (p=0.050) and eating chocolate while drinking coffee (p=0.034). Increased odds of consumption of Arabic coffee were predicted among female, married, overweight, and those who were eating dates while drinking Arabic coffee.

Conclusion

This study concluded that excessive consumption of Arabic coffee was predicted to have a direct association with obesity specifically in female and when mixed with additives including milk, cardamom, and other calorie additives. Furthermore, the odds of obesity tend to increase more when eating chocolate and dates along with coffee. Awareness campaigns are necessary to educate the community about the detrimental effect of unwarranted consumption of Arabic coffee mixed with food or additives.

## Introduction

Obesity is linked to several non-communicable diseases, including diabetes, cardiovascular disease, and cancer [1,2]. It is becoming more common in both developed and developing countries [3]. Body mass index (BMI) is defined as a person's weight in kilograms divided by the square of his or her height in meters (kg/m2). A BMI of 25 to 29.9 is considered overweight, and a BMI of 30 is deemed obese, according to the World Health Organization (WHO) definition of adult obesity [4,5]. There are 155 million overweight children in the world, with 30-45 million categorized as obese [6]. In 2014, 13% of the world's adult population was obese (11% of males and 15% of females), with 39% of adults being overweight [6]. According to the National Nutrition Survey from 2007, the prevalence of obesity in Saudi Arabia was 23.6% in women and 14% in men, with 30.7% of men and 28.4% of women being overweight in the community [7]. Family history, food history and behaviors, diabetes, physical inactivity, sleep disturbance, and genetic variables were all identified as major risk factors for obesity [1].

Coffee is one of the major human sources of caffeine and is considered the most widely consumed beverage worldwide, with a global estimated consumption of more than 2.25 billion cups/per day. Coffee is considered a rich source of caffeine, antioxidants, and anti-inflammatory compounds [8,9]. It is a complex chemical mixture that contains hundreds of biologically active compounds including carbohydrates, lipids, nitrogenous compounds, vitamins, minerals, alkaloids, and phenolic compounds. The average daily caffeine consumption in United States (US) adults is estimated to be 180-190 mg of caffeine, equivalent to two to three cups of coffee [10]. Arabic coffee bean accounts for over 60% of the worldwide produced coffee; it contains a higher lipid content and less caffeine [9]. Traditional Arabic coffee is made of mixed cardamom with dry coffee beans. Cloves and saffron are the two most common additives [1]. Arabic coffee is the mainstay drink in most Saudi homes, served to guests and on local social occasions [11]. Arabic coffee is served from a “dallah," a traditional coffee pot, into a "finjan," a small specialized traditional cup. However, the effect of Arabic coffee intake on health is still controversial. A study has shown that in healthy coffee consumers, the total serum cholesterol was higher than among non-consumers, and the difference was greater among females than among males [9]. Researchers found that in individuals consuming caffeine later in the day, there is a significant increase in sleep onset latency, decreased sleep quality, and decreased total sleep time, these effects were more common among adolescents as they consumed caffeine throughout the day [9].

There were several studies done in various regions of Saudi Arabia about coffee drinking in the general population and the risks associated with it, however, only limited studies are available regarding Arabic coffee specifically. A study was done in Saudi Arabia to find the possible relationship between cholesterol levels and Arabic coffee intake, and it found that cholesterol level is high in coffee drinkers [12]. Another study was performed by Al-Mssallem among 10 healthy subjects who ate Khulas dates with water or Arabic coffee for four months, resulting in increased plasma glucose among those who drink Arabic coffee than those who drink water [13]. In addition, a cross-sectional study conducted among 384 females in Madinah, Kingdom of Saudi Arabia (KSA), found that those who drink too much Arabic coffee have an increased risk of obesity [1].

There are limited data concerning the relationship between Arabic coffee consumption and the risk of obesity between men and women in the population of the Eastern Province, Saudi Arabia. To date, the previous studies investigated the prevalence of Arabic coffee consumption [10], the effect of Arabic coffee on insulin sensitivity [13], and the Arabic coffee-induced risk of obesity among females only [1]. To our knowledge, there is no study on the effect of coffee consumption on the risk of obesity for both genders among the population in the Eastern Province of Saudi Arabia. Hence, this study aims to identify the relationship between Arabic coffee consumption and the risk of obesity.

## Materials and methods

Study population

A randomized cross-sectional study was carried out in the Eastern Province region of Saudi Arabia from January 2022 to April 2022. The questionnaire was obtained from a prior published investigation, in which it was validated [1]. The questionnaire was modified for the purpose of this study. The sample size was estimated at 385, calculated using Raosoft software. The margin of error was determined as 5%, the confidence level as 95%, and a response distribution of 50%. The study included males and females aged above 18 who lived in the Eastern Province. Participants who were pregnant, lactating, with chronic diseases like diabetes, hypertension, coronary artery disease, thyroid disease, and polycystic ovarian syndrome, or on medications that affect weight status such as corticosteroids, anti-depressants, anti-psychotics, and epilepsy medications were excluded from the study. The study's ethical approval was given by the scientific committee of King Faisal University (number KFU-REC-2021- DEC-EA000252).

Data collection

The data was collected through a structured questionnaire. It was formulated in Arabic and was completed using Google Documents. and distributed online via social media applications. Consent was taken from the participants and the privacy of their information was ensured. The questionnaire comprised three main parts. The first part was sociodemographic characteristics (gender, age, marital status, smoking habits, and exercise habits) and anthropometrics, including weight and height. For this, body mass indexes were calculated. Measurements were determined according to the National Institutes of Health (NIH), the BMI classified as underweight (BMI < 18.5), normal (BMI = 18.5- 24.9), overweight (BMI = 25-29.9), or obese (BMI ≥ 30) [12]. The second part assessed the medical status of the participant, and it involved questions about pregnancy, lactation, medications use, and chronic diseases. The last part was directed to explore the consumption of Arabic coffee and the factors associated with it in our participants. It included seven questions that assessed the frequency, amount, timing of coffee consumed per day, and if any additives or foods are being consumed along with it. The last two questions were directed to assess the perception of our participants, whether they think that coffee decreases their appetite or helps them skip meals.

Statistical analysis

SPSS for Windows, version 26.0 (IBM Corp., Armonk, NY, USA) was used to analyze all the data in this project. Demographic data were categorized to calculate numbers and percentages. Chi-square test was used to determine the relationship between the habits of consumption of Arabic coffee and the socio-demographic characteristics of participants. Significant results were then placed in a multivariate regression model to determine the independent significant factor associated with more consumption of coffee with a corresponding odd ratio and 95% confidence interval. We also performed Chi-square test to compare Arabic coffee-induced risk of obesity and gender. A p-value of <0.05 was taken as significant.

## Results

In total, 389 adult Arabic coffee drinkers were recruited. Table 1 presents the socio-demographic characteristics of the participants. More than half were females (57.1%) with nearly 60% being married. Smoking participants were 10.5% while regular exercise was reported by 46.5% of respondents. Approximately one-third of participants (33.7%) were obese while 29.8% were overweight.

**Table 1:** Socio-demographic characteristics of participants living in the Eastern Province, Kingdom of Saudi Arabia (n=389)

Study variables	N (%)
Gender	
Male	167 (42.9%)
Female	222 (57.1%)
Are you over 18 years old?	
Yes	389 (100%)
No	0
Marital status	
Single	157 (40.4%)
Married	232 (59.6%)
Are you smoker?	
Yes	41 (10.5%)
No	348 (89.5%)
Exercise	
Yes	181 (46.5%)
No	208 (53.5%)
Are you from Eastern province?	
Yes	389 (100%)
No	0
BMI level	
Underweight (<18.5 kg/m2)	17 (04.4%)
Normal (18.5 – 24.9 kg/m2)	125 (32.1%)
Overweight (25 – 29.9 kg/m2)	116 (29.8%)
Obese (≥30 kg/m2)	131 (33.7%)

Regarding the attitude of participants toward the consumption of Arabic coffee (Table 2), we observed that 53.5% of participants were drinking coffee daily, in two to three cups (34.4%) and usually during the evening (59.9%). The most common foods eaten along with Arabic coffee by multiple response answers were dates (56.3%) and chocolates (52.2%). Only 19% believed that Arabic coffee decreased appetite during the day and only 17% believed that drinking coffee helps to skip the main meals.

**Table 2:** Attitude of participants toward the consumption of Arabic coffee (n=389) * Variable with multiple response answers.

Variables	N (%)
Frequency of drinking coffee	
Daily	208 (53.5%)
Weekly	145 (37.3%)
Monthly	36 (09.3%)
How much do you drink per day?	
1 cup (30 ml)	52 (13.4%)
2-3 cups (60-90ml)	134 (34.4%)
≥ 4 cups (≥ 120ml)	107 (27.5%)
I don’t drink coffee daily	96 (24.7%)
What time do you usually drink Arabic coffee?	
Morning	83 (21.3%)
Afternoon	73 (18.8%)
Evening	233 (59.9%)
What do you usually eat with Arabic coffee? *	
Chocolates	203 (52.2%)
Dates	219 (56.3%)
I don’t eat anything	31 (08.0%)
Others	27 (06.9%)
Do you think Arabic coffee decreases your appetite during the day?	
Yes	74 (19.0%)
No	230 (59.1%)
I don’t know	85 (21.9%)
Do you think drinking coffee helps you to skip the main meals?	
Yes	66 (17.0%)
No	265 (68.1%)
I don’t know	58 (14.9%)

**Figure 1:**
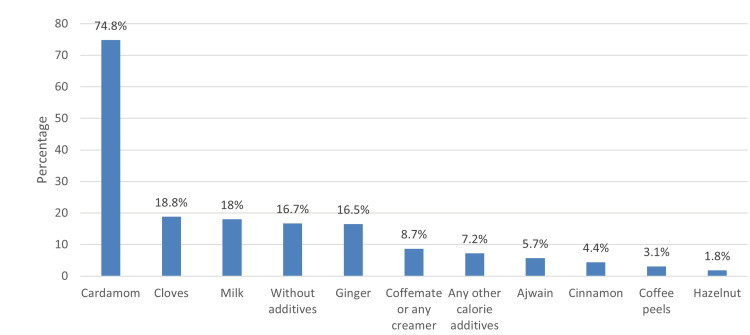
Most favorite additives for coffee

In Figure 1, the most popular additive for coffee was cardamom (74.8%), followed by cloves (18.8%) and milk (18%), while hazelnut was the least popular (1.8%).

We used the Chi-square test in Table 3 to determine the relationship between the consumption of Arabic coffee and the socio-demographic characteristics of participants. Based on the results, it was found that the consumption of Arabic coffee was significantly related to marital status (p<0.001), BMI level (p<0.001), the usual time when drinking Arabic coffee (p<0.001), and eating dates with Arabic coffee (p<0.001). Other variables included in the test did not show a significant relationship with the consumption of Arabic coffee including gender, smoking status, exercise, the perception that Arabic coffee decreases appetite, and the perception that drinking coffee helps to skip the main meals (p>0.05).

**Table 3:** Relationship between the consumption of Arabic coffee and the socio-demographic characteristics of participants (n=389) * Variable with multiple response answers. § P-value has been calculated using Chi-square test. ** Significant at p<0.05 level.

Factor	Consumption of drinking coffee		P-value ^§^
	Everyday N (%) (n=208)	Weekly/Monthly N (%) (n=181)	
Gender			
Male	89 (42.8%)	78 (43.1%)	0.952
Female	119 (57.2%)	103 (56.9%)	
Marital status			
Single	58 (27.9%)	99 (54.7%)	<0.001 **
Married	150 (72.1%)	82 (45.3%)	
Are you a smoker?			
Yes	24 (11.5%)	17 (09.4%)	0.492
No	184 (88.5%)	164 (90.6%)	
Exercise			
Yes	100 (48.1%)	81 (44.8%)	0.512
No	108 (51.9%)	100 (55.2%)	
BMI level			
Normal or underweight	58 (27.9%)	84 (46.4%)	<0.001 **
Overweight	62 (29.8%)	54 (29.8%)	
Obese	88 (42.3%)	43 (23.8%)	
What time do you usually drink Arabic coffee?			
Morning	66 (31.7%)	17 (09.4%)	<0.001 **
Afternoon	40 (19.2%)	33 (18.2%)	
Evening	102 (49.0%)	131 (72.4%)	
What do you usually eat with Arabic coffee? *			
Chocolates	99 (47.6%)	104 (57.5%)	0.052
Dates	136 (65.4%)	83 (45.9%)	<0.001 **
I don’t eat anything	16 (07.7%)	15 (08.3%)	0.829
Others	12 (05.8%)	15 (08.3%)	0.330
Do you think Arabic coffee decreases your appetite during the day?			
Yes	43 (20.7%)	31 (17.1%)	0.108
No	128 (61.5%)	102 (56.4%)	
I don’t know	37 (17.8%)	48 (26.5%)	
Do you think drinking coffee helps you to skip the main meals?			
Yes	40 (19.2%)	26 (14.4%)	0.144
No	143 (68.8%)	122 (67.4%)	
I don’t know	25 (12.0%)	33 (18.2%)	

When conducting multivariate regression estimates (Table 4), it was revealed that being married, overweight, drinking Arabic coffee in the afternoon, and eating dates while drinking Arabic coffee were the independent significant predictors of frequent consumption of Arabic coffee. This further indicates that compared to single, the odds of married participants drinking Arabic coffee more frequently were 2.3 times higher (AOR=2.320; 95% CI=1.437 - 3.747; p=0.001). Respondents who were overweight had a 1.8-fold higher chance of drinking Arabic coffee more frequently than the respondents who were normal/underweight (AOR=1.848; 95% CI=1.050 - 3.254; p=0.003) while the odds of participants who were eating dates while drinking Arabic coffee were predicted to increase the chance of drinking coffee more frequently by at least 1.76 times higher than those who were drinking Arabic coffee alone (AOR=1.757; 95% CI=1.127 - 2.739; p=0.013). On the other hand, compared to participants who usually drank Arabic coffee in the morning, the odds of participants drinking Arabic coffee more frequently were likely to decrease by at least 80% among those who usually drank Arabic coffee in the afternoon (AOR=0.229; 95% 0.123 - 0.425, p<0.001).

**Table 4:** Predictors of frequent Arabic coffee consumption by multivariate regression (n=389) AOR – Adjusted Odds Ratio; CI – Confidence Interval. ** Significant at p<0.05 level.

Factor	AOR	95% CI	P-value
Marital status			
Single	Ref		
Married	2.320	1.437 – 3.747	0.001 **
BMI level			
Normal or underweight	Ref		
Overweight	1.848	1.050 – 3.254	0.033 **
Obese	1.414	0.813 – 2.459	0.220
What time do you usually drink Arabic coffee?			
Morning	Ref		
Afternoon	0.229	0.123 – 0.425	<0.001 **
Evening	0.717	0.409 – 1.258	0.246
Eating dates while drinking Arabic coffee			
No	Ref		
Yes	1.757	1.127 – 2.739	0.013 **

In Table 5, we show the relationship between the level of BMI and Arabic coffee consumption habits according to gender. In the overall participants, it was found that the level of BMI showed a significant relationship with the frequency of drinking coffee (p<0.001) and the number of cups drank per day (p=0.006). BMI was also significantly related to the additives used with coffee such as milk (p=0.017), cardamom (p=0.017), and other calorie additives (p=0.050). Similarly, eating chocolate with coffee showed a significant association with the level of BMI (p=0.034). None of the drinking habits variables showed a significant relationship with the level of BMI in males (all p>0.05). However, females’ levels of BMI have a significant relationship with the frequency of coffee consumption and the addition of additives. Overweight participants’ findings are not displayed due to the lack of significance.

**Table 5:** Relationship between coffee consumption determinants and BMI according to gender * Variable with multiple response answers. § P-value has been calculated using Chi-square test. ** Significant at p<0.05 level.

Factor	Level of BMI in all participants (n=389)			Level of BMI in males (n=167)			Level of BMI in females (n=222)		
	Normal N(%) (n=142)	Obese N(%) (n=131)	P-value ^§^	Normal N (%) (n=56)	Obese N (%) (n=64)	P-value ^§^	Normal N(%) (n=86)	Obese N (%) (n=67)	P-value ^§^
Frequency of drinking coffee									
Daily	58 (40.8%)	88 (67.2%)		26 (46.4%)	39 (60.9%)		32 (37.2%)	49 (73.1%)	
Weekly	63 (44.4%)	38 (29.0%)	<0.001 **	23 (41.1%)	21 (32.8%)	0.436	40 (46.5%)	17 (25.4%)	<0.001 **
Monthly	21 (14.8%)	05 (03.8%)		07 (12.5%)	04 (06.3%)		14 (16.3%)	01 (01.5%)	
How much do you drink per day?									
1 cup (30 ml)	21 (14.8%)	16 (12.2%)		07 (12.5%)	09 (14.1%)		14 (16.3%)	07 (10.4%)	
2-3 cups (60-90ml)	47 (33.1%)	48 (36.6%)	0.006 **	18 (32.1%)	26 (40.6%)	0.128	29 (33.7%)	22 (32.8%)	0.105
≥ 4 cups (≥ 120ml)	27 (19.0%)	48 (36.6%)		12 (21.4%)	22 (34.4%)		15 (17.4%)	26 (38.8%)	
Favorite additives for coffee *									
Milk	35 (24.6%)	15 (11.5%)	0.017 **	12 (21.4%)	08 (12.5%)	0.333	23 (26.7%)	07 (10.4%)	0.043 **
Cardamom	95 (66.9%)	101 (77.1%)	0.017 **	35 (62.5%)	44 (68.8%)	0.306	60 (69.8%)	57 (85.1%)	0.021 **
Ginger	23 (16.2%)	22 (16.8%)	0.991	09 (16.1%)	10 (15.6%)	0.980	14 (16.3%)	12 (17.9%)	0.946
Cloves	20 (14.1%)	28 (21.4%)	0.200	04 (07.1%)	11 (17.2%)	0.110	16 (18.6%)	17 (25.4%)	0.601
Cinnamon	10 (07.0%)	06 (04.6%)	0.054	02 (03.6%)	04 (06.3%)	0.541	08 (09.3%)	02 (03.0%)	0.016 **
Any other calorie additives	08 (05.6%)	06 (04.6%)	0.050 **	04 (07.1%)	02 (03.1%)	0.450	04 (04.7%)	04 (06.0%)	0.062
Without additives	28 (19.7%)	23 (17.6%)	0.248	14 (25.0%)	14 (21.9%)	0.774	14 (16.3%)	09 (13.4%)	0.235
Usually eaten with coffee *									
Chocolate	84 (59.2%)	57 (43.5%)	0.034 **	27 (48.2%)	22 (34.4%)	0.279	57 (66.3%)	35 (52.2%)	0.212
Dates	72 (50.7%)	83 (63.4%)	0.104	33 (58.9%)	48 (75.0%)	0.171	39 (45.3%)	35 (52.2%)	0.697
I don’t eat anything	12 (08.5%)	14 (10.7%)	0.175	03 (05.4%)	02 (03.1%)	0.708	08 (09.3%)	02 (03.0%)	0.106

## Discussion

The present study examined Arabic coffee consumption and its relation to obesity among the Saudi general population living in the Eastern Province. The findings of this study revealed that daily drinking of Arabic coffee was identified among 53.5% of the population. Of them, 33.7% were obese and 29.8% were overweight and the prevalence of obesity among coffee drinkers was significantly higher. Furthermore, our study also revealed that drinking Arabic coffee with additives along with eating chocolate as well as dates increased the risk of being obese. In Madinah, Saudi Arabia, a similar study was conducted among the Saudi female population, where they discovered that 71.4% consumed Arabic coffee daily and a high level of coffee consumption was directly associated with obesity among Saudi female coffee drinkers [10]. These findings are in agreement with the study done in Makkah, Saudi Arabia [11], by Rezq et al. who suggested that people who consumed low amounts of Arabic coffee were predicted to have no significant increase in BMI level and blood pressure as compared to those who consumed high amount. A study from Korea revealed a similar relationship between obesity and coffee consumption [14,15]. On the other hand, a Canadian study showed that the frequency of coffee consumption alone has no association with measures of obesity, but the additives used with it have been shown to have a direct relation to BMI and waist circumference [16]. Similarly, in this study, the risk of obesity when drinking Arabic coffee alone was less since the component of caffeine was minimal compared to other kinds of coffee [17]. Thus, the increase in BMI level could be due to the additives and food consumption along with Arabic coffee [17].

There are several factors linked to Arabic coffee which elucidate its influence on the increased risk of obesity. For instance, more than one-third (34.4%) were drinking two to three cups or four cups or more (27.5%) of coffee per day. Also, approximately 60% usually drank coffee at night mixed with cardamom (74.8%), cloves (18.8%), or milk (18%) and simultaneously ate with dates (56.3%) or chocolates (52.2%). This pattern of coffee consumption could lead to an increase in weight. This scenario explained the direct correlation between the consumption of Arabic coffee and the risk of obesity. These findings are almost in agreement with the study published in Makkah, Saudi Arabia [11]. According to their reports, daily consumption of Arabic coffee was reported by 62.5% of the population, two to three cups (37.5%) to four or more cups (37.5%), however, half of them drank coffee in the afternoon (50%), higher than those who drank at night (37.5%) with mostly cardamom (97.5%) and ginger (70%) as additives. Supporting these reports, a study published in Sweden [18] implicated that drinking coffee at night may lead to obesity [18].

Frequent consumption of Arabic coffee was significantly predicted among the married and overweight population. It may be true that married people tended to consume more coffee compared to single ones, due to their responsibility to family or work. These views may be consistent with that of Lee et al. [14]. In their report, participants with the highest coffee consumption tended to be younger, have more education, have lower unemployment rates, be current drinkers, be current smokers and exercise less regularly. Another study published in Poland [19] indicated that a higher prevalence of coffee and tea consumers was found among the female gender, young age, and smokers. Moreover, high consumption was found in people with medium-high educational level, with high total energy intake. Surprisingly, in their study, they concluded that high coffee and tea consumption were associated with a decreased prevalence of central obesity and better cholesterol and glucose metabolism. This may be due to the disparity in the components of coffee where coffee containing polyphenols was the most common coffee among Polish people.

None of the habitual factors of Arabic coffee consumption were significant to the level of BMI in men, however, in women, the level of BMI was significant to frequent consumption of Arabic coffee, along with additives such as milk, cardamom, and cinnamon. These findings are almost consistent with the study done in Canada [16]. This study found that coffee consumption was not related to BMI or waist circumference in both genders, and they concluded that the frequency of coffee/tea consumption did not prevail significantly with measures of obesity because the ingredients used explained the causation between tea consumption and obesity in men. Another study conducted among Japanese Civil Servants [20] discovered that high blood pressure and high triglyceride level were inversely associated with moderate coffee consumption in men. However, in women, moderate coffee consumption was not significantly associated with the prevalence of metabolic syndrome or its components and they ultimately found no difference in BMI of both genders in moderate coffee consumption.

A few limitations should be taken into consideration while interpreting the findings of this research. First, the sample size is not representative of Saudi Arabia's overall adult population. Second, this is a cross-sectional study, hence, no causation or effect can be obtained. Moreover, the anthropometric measurement information was self-reported which could have led to recall bias.

Nevertheless, this study also has several strengths, such as the inclusion of participants of both genders, different body compositions, and ages. Furthermore, based on our information, this study is a novel contribution to the literature in comparing the association of coffee consumption and various sociodemographic variables, consumption determinants, and associated food habits between males and females.

## Conclusions

This study concluded that excessive consumption of Arabic coffee was predicted to have a direct association with obesity specifically in females and when mixed with additives including milk, cardamom, and other calorie additives. Furthermore, the odds of obesity tend to increase more when eating chocolate or dates along with coffee. Awareness campaigns are necessary to educate the community about the detrimental effect of unwarranted consumption of Arabic coffee mixed with food or additives. The findings of this study highlight the risk of obesity due to frequent drinking of Arabic coffee which needed further research to establish its true relations. A future large-scale study on a nationwide level that could verify the true causal relationship between the consumption of Arabic coffee and its relation to obesity in the general population of Saudi Arabia is further recommended.

## References

[1] Jalloun RA, Alhathlool MH (2020). Arabic coffee consumption and the risk of obesity among Saudi’s female population. J Saud Soc Food Nutr.

[2] NHLBI Obesity Education Initiative Expert Panel on the Identification, Evaluation Evaluation, and Treatment of Obesity in Adults (US) (1998). Clinical Guidelines on the Identification, Evaluation, and Treatment of Overweight and Obesity in Adults: The Evidence Report. Obes.

[3] Flegal KM, Graubard BI, Williamson DF, Gail MH (2005). Excess deaths associated with underweight, overweight, and obesity. JAMA.

[4] (2016). Obesity and overweight. http://www.who.int/mediacentre/factsheets/fs311/en/.

[5] (2016). Body mass index (BMI). https://www.who.int/data/gho/data/themes/topics/topic-details/GHO/body-mass-index?introPage=intro_3.html.

[6] Mirmiran P, Sherafat-Kazemzadeh R, Jalali-Farahani S (2010). Childhood obesity in the Middle East: a review. East Mediterr Health J.

[7] Alwasaidi TA, Alrasheed SK, Alhazmi RA, Alfraidy OB, Jameel MA, Alandijani AA (2017). Relation between ABO blood groups and obesity in a Saudi Arabian population. J Taibah Univ Med Sci.

[8] Buscemi S, Verga S, Batsis JA (2009). Dose-dependent effects of decaffeinated coffee on endothelial function in healthy subjects. Eur J Clin Nutr.

[9] AlShareef S (2021). Caffeine extraction from Arabic coffee: the role of brewing and roasting. Imam J Appl Sci.

[10] Alfawaz HA, Khan N, Yakout SM (2020). Prevalence, predictors, and awareness of coffee consumption and its trend among Saudi female students. Int J Environ Res Public Health.

[11] Rezq AA, Qadhi AH, Almasmoum HA, Ghafouri KJ (2021). Effect of Arabian coffee (Saudi coffee) consumption on body mass index, blood glucose level and blood pressure in some people of Makkah Region, KSA. Rev Kasmera.

[12] el Shabrawy Ali M, Felimban FM (1993). A study of the impact of Arabic coffee consumption on serum cholesterol. J R Soc Health.

[13] Al-Mssallem MQ, Brown JE (2013). Arabic coffee increases the glycemic index but not insulinemic index of dates. Saudi Med J.

[14] Lee J, Kim HY, Kim J (2017). Coffee consumption and the risk of obesity in Korean women. Nutrients.

[15] Kim JH, Park YS (2017). Light coffee consumption is protective against sarcopenia, but frequent coffee consumption is associated with obesity in Korean adults. Nutr Res.

[16] Bouchard DR, Ross R, Janssen I (2010). Coffee, tea and their additives: association with BMI and waist circumference. Obes Facts.

[17] Naser LR, Sameh A, Muzaffar I, Omar AR, Ahmed MA (2018). Comparative evaluation of caffeine content in Arabian coffee with other caffeine beverages. Afr J Pharm Pharmacol.

[18] Bertéus Forslund H, Lindroos AK, Sjöström L, Lissner L (2002). Meal patterns and obesity in Swedish women-a simple instrument describing usual meal types, frequency and temporal distribution. Eur J Clin Nutr.

[19] Grosso G, Stepaniak U, Micek A, Topor-Mądry R, Pikhart H, Szafraniec K, Pająk A (2015). Association of daily coffee and tea consumption and metabolic syndrome: results from the Polish arm of the HAPIEE study. Eur J Nutr.

[20] Matsuura H, Mure K, Nishio N, Kitano N, Nagai N, Takeshita T (2012). Relationship between coffee consumption and prevalence of metabolic syndrome among Japanese civil servants. J Epidemiol.

